# Assessment of a novel smartglass-based point-of-care fusion approach for mixed reality-assisted targeted prostate biopsy: A pilot proof-of-concept study

**DOI:** 10.3389/fsurg.2022.892170

**Published:** 2022-07-22

**Authors:** P. Sparwasser, M. Haack, L. Frey, K. Boehm, C. Boedecker, T. Huber, K. Stroh, M. P. Brandt, R. Mager, T. Höfner, I. Tsaur, A. Haferkamp, H. Borgmann

**Affiliations:** ^1^ Department of Urology, University Medical Center Johannes Gutenberg University, Mainz, Germany; ^2^ Department of General, Visceral and Transplant Surgery, University Medical Center Johannes Gutenberg University, Mainz, Germany; ^3^ Department of Radiology, University Medical Center Johannes Gutenberg University, Mainz, Germany; ^4^ Department of Urology, Brandenburg Medical School Theodor Fontane, Neuruppin, Germany

**Keywords:** prostate biopsy, mixed reality, augmented reality, hololens, prostate cancer, smartglass

## Abstract

**Purpose:**

While several biopsy techniques and platforms for magnetic resonance imaging (MRI)-guided targeted biopsy of the prostate have been established, none of them has proven definite superiority. Augmented and virtual reality (mixed reality) smartglasses have emerged as an innovative technology to support image-guidance and optimize accuracy during medical interventions. We aimed to investigate the benefits of smartglasses for MRI-guided mixed reality-assisted cognitive targeted biopsy of the prostate.

**Methods:**

For prospectively collected patients with suspect prostate PIRADS lesions, multiparametric MRI was uploaded to a smartglass (Microsoft® Hololens I), and smartglass-assisted targeted biopsy (SMART TB) of the prostate was executed by generation of a cognitive fusion technology at the point-of-care. Detection rates of prostate cancer (PCA) were compared between SMART TB and 12-core systematic biopsy. Assessment of SMART-TB was executed by the two performing surgeons based on 10 domains on a 10-point scale ranging from bad (1) to excellent (10).

**Results:**

SMART TB and systematic biopsy of the prostate were performed for 10 patients with a total of 17 suspect PIRADS lesions (PIRADS 3, *n* = 6; PIRADS 4, *n *= 6; PIRADS 5, *n* = 5). PCA detection rate per core was significant (*p* < 0.05) higher for SMART TB (47%) than for systematic biopsy (19%). Likelihood for PCA according to each core of a PIRADS lesion (17%, PIRADS 3; 58%, PIRADS 4; 67%, PIRADS 5) demonstrated convenient accuracy. Feasibility scores for SMART TB were high for practicality (10), multitasking (10), execution speed (9), comfort (8), improvement of surgery (8) and image quality (8), medium for physical stress (6) and device handling (6) and low for device weight (5) and battery autonomy (4).

**Conclusion:**

SMART TB has the potential to increase accuracy for PCA detection and might enhance cognitive MRI-guided targeted prostate biopsy in the future.

## Introduction

Since the first version for PI-RADS in 2011, multiparametric magnetic resonance imaging (mpMRI) of the prostate for diagnosis of prostate cancer (PCA) has been widely adopted into daily routine. While execution for mpMRI is strictly defined, the technique for subsequent targeted prostate biopsy in case of PCA suspicion is not determined. To date several techniques for targeted prostate biopsy have been developed: in-bore MRI biopsy (MRI-TB), software fusion biopsy (FUS-TB) and cognitive fusion biopsy (COG-TB). All of them demonstrated to have approximately comparable detection rates for PCA (1). To date there is still no definite recommendation for one of these techniques (2) and therefore the discourse on how best to perform targeted biopsy continues (1–4).

Mixed reality tools proofed to facilitate the real-time integration of medical data and radiological imaging into surgical procedures (5, 6) and are thus increasingly evaluated for use in clinical practice (7). Thereby, intraoperative surgical applications for smartglasses may offer great opportunities by real-time overlay of preoperative imaging at the point-of-care (5, 7) and demonstrated to be feasible and safe (6). Most of these technical applications consist of a head-mounted display integrated to a smartglass with a see-through display for mixed reality-assisted surgery, allowing its user to execute surgery under visualization of virtual imaging while an unobstructed view to the operation field is given.

In PCA diagnosis and treatment, several virtual and augmented reality applications using smartglasses have currently been developed (7, 8). Within a pilot proof-of-concept study, we already investigated the possible benefits in using a smartglass (Vuzix Blade®, Rochester USA) for augmented reality assisted prostate biopsy (9). Here we demonstrated good feasibility and convenient detection rates but also stated the necessity of hardware enhancements especially according to image quality and the need of further prospective investigations (9). Following up on our pilot proof-of-concept study, we aimed to transfer our first experiences with SMART TB onto a lager cohort and to a more advanced mixed reality tool.

This is the first report on using cognitive point-of-care fusion technology for mixed reality smartglasses-assisted targeted biopsy (SMART TB) under usage of the smartglass Hololens (Microsoft®) for prostate cancer diagnosis.

## Methods

**Figure 1:**
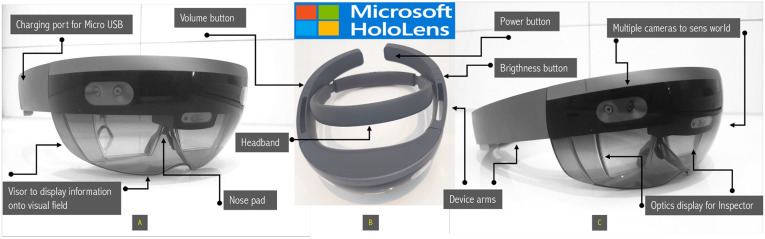
Hololens I by Microsoft® is a see-through smartglass with holographic lenses which creates 3-D models into the surrounding environment due to multiple spatial-mapping cameras and depth cameras, as well its inertial measurement unit. Navigation: eye tracking, finger tracking, voice commands. Processor: Holographic Processing Unit HPU 1.0 Intel 32-bit with Operating System Windows 10+ Windows Store; Memory: 2 GB RAM, 64 GB Flash Storage; Weight 759 g; Camera 2.4 MP photo; 1.1 MP HD video, video speed 30 FPS; Batery life 2–3 h under active use and 2 weeks standby; Connectivity to other sources through Wi-Fi 802.11ac, Bluetooth 4.1 LE and Micro-USB 2.0 (11). Figure 2 illustrates hardware components of Hololens I from (**A**) front, (**B**) side and (**C**) bird’s eye view.

Ten patients were included prospectively in our feasibility study after approvement by the local ethics board. Inclusion criteria were suspect for prostate cancer by moderate PSA elevation (≤20 ng/ml) and by mpMRI imaging with a maximum of three target lesions per patient while maximum size of the prostate was set at ≤120 ml. In accordance with the EAU guidelines preoperative mpMRI imaging was executed to avoid unnecessary biopsy in asymptomatic men with moderate PSA elevation (2). All patients underwent mpMRI (1.5- or 3-Tesla) of the prostate with confirmation of a minimum of one suspect prostate lesion. A genitourinary expert of local radiology department reviewed each mpMRIs and external imaging was filtered through quality checks by same genitourinary expert, while a maximum of two targets were labelled for each patient. Based on Diffusion-weighted, T2-weighted and contrast-enhanced series, MRI lesions were given in accordance to current standardization criteria a *Prostate Imaging Reporting and Data System score* (PI-RADSv2) from 1 to 5 (10). As previous described through our study group for the usage of the smartglass Vuzix® Blade we processed the mpMRI scans to produce a two-dimensional image copy including the standardised mpMRI reporting scheme with labelled index lesion, relevant axial T2-weighted images (base/mid/apex) with demonstrative landmarks and the afore labelled target lesion/lesions (9). These data were uploaded via micro-USB 2.0 now to the smartglass Microsoft® Hololens I (Figure 1) prior to biopsy as a JPEG file.

**Figure 2:**
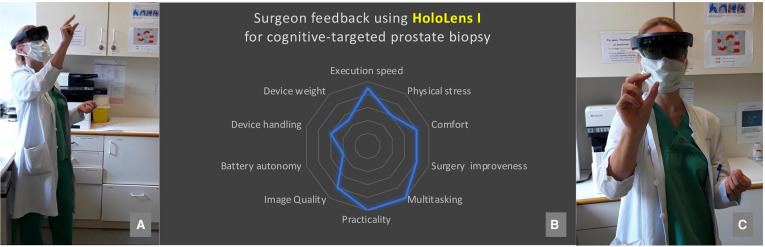
Illustration of surgeon wearing Hololens smartglass and navigation by finger tracking [picture (**A,C**)]. Surgeons’ feedback for using Hololens I for cognitive-targeted prostate biopsy including an assessment from bad to excellent for specific domains [picture (**B**)].

Targeted biopsy of the prostate was indicated in the presence of lesions with PIRADS scores between 3 and 5 in accordance to the EAU Guidelines (2). Thus, we choosed an ultrasound-guided transrectal approach using the HiVision Ascendus Ultrasound (Hitachi Medical Systems®). A preoperative application of intravenous antibiotics (ceftriaxon or ciprofloxacin) and rectal disinfection (povidone-iodine) was additionally executed. Patients’ tolerance was improved by infiltration of the periprostatic plexus with local anaesthesia (mecain 2%) as it is described widely. In the meantime, while the patient still placed in lithotomy position, the uploaded mpMRI files were retrieved from the mixed reality smartglass Hololens I (Figure 2). Through the smartglass the biopseur was now enabled to create a mixed reality operating room at the point-of-care by attaching all previously uploaded MRI files including the prostate scheme of the prostate as holographic projection around the patient (still in lithotomy position).

Navigation of the Hololens was thereby possible hands-free through voice commands and finger tracking (Figure 2) under aseptic conditions. Finger tracking thereby describes the possibility of the smartglass to recognized and process user’s manual interaction with the projected virtual data. Target biopsy was thereafter performed by two experienced surgeons with a transrectal software fusion biopsy case load of each more than 100 normally using the Hi-RVS Preirus-System for the HiVision Ascendus Ultrasound (Hitachi Medical Systems®). Our SMART TB was then always followed by a systematic transrectal 12-core biopsy. SMART TB was compared to the concomitant executed 12-core systematic biopsy (by same biopseur) and to the abovementioned well-established MRI-based biopsy techniques. In summary, we obtained between 16 and 22 samples per patient (target biopsy, 4–10 cores; systematic biopsy, 12 cores). The number of cores taken depended on surgeon’s assessment taking lesion count and size in to account. As already prescribed in our pilot-proof of concept study the surgeon cognitively matched for targeted biopsy the real-time transrectal ultrasound with the uploaded MRI images and optimization of accuracy was possible through hand-guided adjustment of the puncture line according to the specific landmarks on mpMRI images displayed in front of surgeon’s eyes (9). In the meanwhile, the surgery field remained unrestricted and cognitive matching between MRI images and real-time ultrasound at the point-of-care was enabled through view-switching to display the holographic projection into the field of vision.

Examination of the separately enumerated biopsy cores was executed through a designated uropathologist expert. Assessment of SMART TB using the smartglass Hololens I by the two performing surgeons was based on following criteria (1 = bad to 10 = excellent) and ten domains adopted from Galati et al.: execution speed, physical stress, comfort, surgery improveness, multitasking, practicality, image quality, battery autonomy, device handling, and device weight (11). Descriptive statistics were used to report patient data, as well operating room times and costs for SMART TB were analyzed, while clinical data were prospectively collected and perioperative complications and outcomes were assessed. Chi-square test was used to compare groups and those results with *p* values <0.05 were consid­ered statistically as significant. Finally, we want to state that this pilot proof-of-concept study aims to assess primarily the feasibility of prostate biopsy using the smartglass Hololens I.

## Results

SMART TB of the prostate was performed in 10 patients (patients A–J) with suspected PCA, while 7 patients undergoing biopsy for the first and 3 for the second time. The average age of the patients was 70.8 years, while mean PSA elevation was 8.4 ng/dl and digital rectal examination with suspicious for PCA was observed in 2 patients (Table 1).

**Table 1:** Patient characterization.

Patient Factors
Parameter, unit		
Mean Age (years)	70.8	(Range 62–79)
ECOG 0	80%	
ECOG 1	20%	
iPSA (ng/ml)	8.4	(Range 4.1–8.9)
PSA ratio	0.18	(Range 0.09–0.31)
PSA density (ng/ml^2^)	0.14	(Range 0.07–0.23)
Prostate volume (ml)	61.2	(Range 32–100)
Suspicious DRE, *n* (%)	2	20%
Previous negative biopsy, *n* (%)	3	30%
Total PIRADS lesions, *n* (%)	17	
PIRADS 3	6	35.30%
PIRADS 4	6	35.30%
PIRADS 5	5	29.40%
Localization PIRADS lesion, *n* (%)	17	
Apex	4	23.50%
Mid	9	52.90%
Base	4	23.50%
	17	
Peripheral zone	13	76.50%
Transitional zone	3	17.65%
Anterior fibromuscular stroma	1	5.90%

According to the intraoperative adverse events classification (EAUiaiC) and postoperative complications classifications (Clavien Dindo), we observed no intraoperative complications and minor postoperative complications (Table 2). The average OR time was ∼32 min for all cases. SMART TB is intended to expand cognitive fusion biopsy. Therefore, compared to a cognitive fusion biopsy no extra cost beside the equipment acquisition for the Hololens I are necessary. Besides general cost for MRI and biopsy equipment, the investment cost for the Microsoft® Hololens I was ∼4580 USD.

**Table 2:** Results of prostate biopsy and histological examination.

Results of Biopsy		
Overall positive Cores, *n* (total cores)	51	180
Positive Cores, %	28.33%	
Positive Cores systematic biopsy, *n* (total cores)	23	120 (12/person)
Positive Cores systematic biopsy, %	19.17%	(Range 0–41.66)
Median positive cores per person	2.3	
Positive Cores SMART TB, *n* (total cores)	28	60 (6/person)
Positive Cores SMART TB, %	46.67%	(Range 0–100)
Median positive cores per person	2.8	
Positive Cores per PIRADS lesion, (%)		
PIRADS 3	16.66%	(0–100)
PIRADS 4	58.33%	(0–100)
PIRADS 5	61.66%	(25–100)
Intraoperative Adverse Events (EAUiaiC)	None	0%
Postoperative complications within 7 days (Clavien Dindo >1)	None	0%
Histological Results		
Prostate cancer, Gleason grade: systematic biopsy vs. SMART TB	Systematic biopsy	SMART TB
Patient A	Gleason 6	None
Patient B	Gleason 6	Gleason 6
Patient C	Gleason 6	Gleason 6
Patient D	Gleason 6	Gleason 6
Patient E	None	None
Patient F	Gleason 7b	Gleason 8
Patient G	Gleason 7b	Gleason 7b
Patient H	None	Gleason 7a
Patient I	Gleason 7a	Gleason 7a
Patient J	PIN	PIN
PCa (Gleason ≥3 + 3* *=* *6), *n* (%)	8	80%
csPCa (Gleason ≥4 + 3* *=* *7*a*), *n* (%)	4	40%
Prostate cancer likelihood per PIRADS lesion, % (per targeted core of PIRADS lesion)		
PIRADS 3	16.67%	(16.67%)
PIRADS 4	66.67%	(58.3%)
PIRADS 5	100.0%	(61.67%)

Table 2 shows the histological results of systematic biopsy in comparison to SMART TB. Overall, 180 cores were obtained and 51 (28%) of these showed PCA of any Gleason score (≥3 + 3 = 6). 28 of 60 cores (47%) for SMART TB and 23 of 120 cores (19%) for systematic biopsy revealed PCA of any kind. The chi-square statistics revealed with a p-value of 0.000114 (*p *< 0.05) a significant superiority for SMART TB. Considering the PI-RADS v2 score, PIRADS lesions (*n *= 17) were distributed over the complete prostate, but they were more likely found in the mid (53%) and peripheral zone (76%). The detection rates for particular PIRADS lesions showed 17% for PIRADS 3 (1/6), 67% for PIRADS 4 (4/6) and 100% for PIRADS 5 (5/5), while detailed analysis demonstrated likelihood for prostate cancer according to each targeted core of a PIRADS lesion was 17% (PIRADS 3), 58% (PIRADS 4) and 62% (PIRADS 5).

Adenocarcinoma of the prostate was found within histological examination in eight (80%) and clinically significant prostate cancer (csPCa) in four (40%) cases regardless of biopsy technique. In addition, only due to SMART TB detection of csPCa was observed in one patient and another patient received upgrading to high risk PCA because of SMART TB, while one low risk prostate cancer (Gleason 3 + 3 = 6) was only detected by systematic biopsy.

The performing surgeon assessed SMART TB using the smartglass Hololen I towards abovementioned criteria (scale from 1 to 10): multitasking (10), practicality (10), execution speed (9), comfort (8), surgery improvement (8), image quality (8), physical stress (6), device handling (6), device weight (5), battery autonomy (4) (Figure 2).

## Discussion

Beside the fact that technical maturity of smartglasses is yet missing (13), several studies demonstrated general feasibility, safety and usefulness of smartglasses in the field urology (6,12). Based on our findings from our initial pilot proof-of-concept study with first description of SMART TB (9) we transferred our promising experiences to a more advanced mixed-reality tool now using Microsoft’s Hololens I and extrapolate our innovative approach towards a larger cohort (*n *= 10). SMART TB using the smartglass Hololens I was expectable associated with higher detection rates then the common 12-core systematic biopsy (47% vs. 19%) for PCA of any kind, while we must clarify that systematic biopsy is not the most appropriate comparator. Histological examination revealed adenocarcinoma of the prostate in 8 of 10 patients (80%), while detection of csPCa was observed in 4 out of 10 cases (40%). These findings are almost overlapping with our results from our pilot-proof-of concept study performing SMART TB using the Vuzix®Blade, were we demonstrated PCA detection rates for all SMART TB of 46%. For regular cognitive MRI-guided biopsy techniques, the literature reports here referring to larger cohorts detection rates between 27.0% and 69.7% for csPCa (9). In further comparison Wegelin et al. demonstrated respectively for the well-established but more sophisticated techniques like in-bore MRI target biopsy (MRI-TB) and MRI-TRUS fusion target biopsy (FUS-TB) detection rates for csPCa of 55% and 49% (1). However, there is some evidence that MRI-TB, neglecting its complex and expensive set-up, achieves superior detection rates compared to FUS-TB and COG-TB (14).

Rouviere et al. reported for example poorer detection rates for targeted biopsy over systematic biopsy (32.3% vs. 29.9%) in 251 men (15) than that reported for SMART TB (47% vs. 19%) in our study. Taking the findings of our pilot-proof-of concept paper into concern we also demonstrated for SMART TB using the Vuzix® Blade higher detection rates over systematic biopsy (46% vs. 27%) (9). However, due to the small cohort size (*n *= 10) in our study we have to state that a suitable comparison with these findings is currently not possible. Furthermore, according to the PI-RADSv2 score classification targeted biopsy for a total of 17 index lesions lead to detection of PCA in 17%, 67% and 100% for PIRADS 3, PIRADS 4 and PIRADS 5 lesions. Literature shows here to be highly inhomogeneous, while Barkovich et al. demonstrated satisfying overall sensitivity for suspected lesions with a PIRADS score ≥3 in their meta-analysis (including 59 studies) with observation of detection rates for csPCa after targeted prostate biopsy of 6% for PIRADS 1/2, 12% for PIRADS 3, 48% for PIRADS 4 and 72% for PIRADS 5 (16).

Summarized, we found higher detection rates using SMART TB than systematic biopsy, and although PCA was often detected by both procedures, its detection was more likely and more accurate due to upgrading when SMART TB was performed. In comparison to common cognitive MRI-guided biopsy techniques, we found no major difference concerning detection rates, even if comparison lack of evidence. In addition, SMART TB using the Hololens I was expectable performed safely for surgeons and patients, while we observed no intraoperative (EAUiaiC) and no major postoperative (Clavien Dindo) adverse events.

Some minor findings also need to be addressed. Even if to date technical maturity is yet missing (13), a rash of three-dimensional visualisation techniques using augmented or virtual reality have been investigated for surgery. These tools especially focus on education, training models, surgical planning and intraoperative guidance (17). Our findings demonstrate good overall operability for SMART TB using Microsoft® Hololens I. Assessment of SMART TB by our two surgeons according to the adopted criteria of Galati et al. (11) revealed good clinical practice in the domains of multitasking, practicality, execution speed, comfort and surgery improvement, while device handling, device weight and battery autonomy still require improvement (Figure 2). It is noteworthy that especially due to enhanced image quality of the Hololens I a relevant improvement of SMART TB was observed, as compared to our pilot proof-of-concept study using the Vuzix® Blade Version 1.0 (9).

However, cognitive real-time matching at point-of-care of transrectal ultrasound with the mpMRI images optimizes orientation and navigation. With future advancements in the field of mixed reality technologies, it can be assumed that optimized intraoperative guidance will lead to superior accuracy. Our results concur with the conclusion of several colleagues that technological improvements are still necessary before mixed reality devices can be used regular in operating rooms (11), as well more studies are needed to clarify their widespread use (17). With further improvements in see-through devices optimizing image quality and the widespread use of software generating easily three-dimensional reconstruction of radiological imaging, augmented and virtual reality application for intraoperative guidance is expected to be widely implemented in clinical practice (7).

Some limitations of this study need to be addressed. First, we must state that comparing systematic biopsy and SMART TB could lead to misinterpretation of superior SMART TB detection rates, especially because both procedures were performed by same physicians. In general, SMART TB as we described above stays to date a simple cognitive target biopsy, even if next stages of technology developments will focus on generation of intraoperative matching tools for smartglasses. Our observed detection rates therefore may have been achieved with common COG-TB without using a smartglass. Additionally, even better detection rates could have been observed using MRI-TB or FUS-TB. Noteworthy, detection rates for MRI-based biopsy techniques including our SMART TB are highly dependent on the experience of the performing surgeons and their ability to understand prostate mpMRI and transrectal ultrasound (18, 19), as well our small cohort size of only ten patients represents certainly a source of bias.

Finally, we aimed to improve pure cognitive matching for target biopsy of the prostate through a mixed reality tool using a smartglass. Based on our findings we believe to have created a feasible innovative procedure that beside the need of further technical developments contains great potential to increase detection rates of csPCa in future.

## Conclusion

SMART TB of the prostate might enhance MRI-guided targeted prostate biopsy and has the potential to increase detection rates of clinical significant prostate cancer in future, even if further investigation and technical developments are still highly warranted.

## Data Availability

The raw data supporting the conclusions of this article will be made available by the authors, without undue reservation.
